# Supportive Built Environment for Active Aging in Korea’s Healthy City

**DOI:** 10.1093/geroni/igaa057.3235

**Published:** 2020-12-16

**Authors:** Eunju Hwang, Chungwen Hsu, Jinwook Jeong, Nancy Brossoie, Kimin Song

**Affiliations:** 1 Virginia Tech, Blacksburg, Virginia, United States; 2 Hanyang University, Seoul, Seoul-t’ukpyolsi, Republic of Korea

## Abstract

Many small and medium-size towns in South Korea experience an increasing number of older adults growing older. This medium-size mountain city has been a member of WHO’s Healthy City Alliances and we investigated the impacts of built environment (BE) in the community on active aging. The purpose of the survey was to identify the BE affecting the residents’ physical activity level and quality of life. The survey data (N=630) were collected from the residents aged 40 years and over in May 2019 from a mountain city near a national park in Northeast part of Korea. The average age of the participants was 67.41 years of age and most wanted to stay in their community. Our descriptive data analysis show that the following items are perceived very important to address in creating more supportive environment: benches in public spaces; safe pathways and public transportation station; well-maintained sidewalks; affordable and accessible housing options. Regression models were also developed to examine the impacts of BE on activity level and quality of life. BE factors included the features related to outdoor spaces and buildings, public transportation, streetscape and housing, and found a significant relation between the participants’ activity level and public transportation in addition to demographic factors such as age and health status. Regarding the quality of life, the participants’ perception on outdoor spaces and buildings and safe public spaces were positively related to quality of life. The study showed the importance of supportive and age-friendly environments for active living.

